# Depression of Bone Density at the Weight-Bearing Joints in Wistar Hannover Rats by a Simulated Mechanical Stress Associated With Partial Gravity Environment

**DOI:** 10.3389/fcell.2021.707470

**Published:** 2021-07-26

**Authors:** Shenke Zhang, Daishin Ueno, Takashi Ohira, Hisashi Kato, Tetsuya Izawa, Sakuya Yamanouchi, Yukari Yoshida, Akihisa Takahashi, Yoshinobu Ohira

**Affiliations:** ^1^Gunma University Heavy Ion Medical Center, Maebashi, Japan; ^2^Graduate School of Science and Technology, Nara Institute of Science and Technology, Ikoma, Japan; ^3^Research Center for Space and Medical Sciences, Doshisha University, Kyotanabe, Japan; ^4^Organization for Research Initiatives and Development, Doshisha University, Kyotanabe, Japan; ^5^Department of Physiology and Regenerative Medicine, Kindai University Faculty of Medicine, Osakasayama, Japan; ^6^Graduate School of Health and Sports Science, Doshisha University, Kyotanabe, Japan

**Keywords:** partial gravity, simulated mechanical stress, bone parameters, distal femur, proximal tibia

## Abstract

The partial gravity environment in space can negatively affect bone health. This survey aimed to study the reaction of different parts of the lower limb bones of rats to partial gravity and the effects of different degrees of gravity on these bony parts. We used 15 8-week-old male Wistar Hannover rats were used at the beginning of the experiment. The degree of mechanical stress was modified, but the ankle joint was maintained at ∼30°, ∼120°, or ∼160° with or without plaster fixation during 10-day hindlimb suspension. Computed tomography was performed to measure the bone parameters [bone mineral density (BMD), trabecular BMD, cortical BMD, and cortical thickness] of each studied group of the whole, proximal, middle, and distal femur and distal tibia. BMD, trabecular BMD, and cortical thickness of the distal femur and proximal tibia of the simulated mechanical stress associated with partial gravity groups were significantly lower than those of the control group; the effect of different degrees of gravity on the same area of hindlimb bone had no significant difference. The simulated mechanical stress associated with partial gravity had the most significant effect on the bone close to the knee joint, with the largest weight-bearing response.

## Introduction

It has been almost 60 years since the Soviet Union launched the first manned spacecraft. In the subsequent decades, humans have conducted various space explorations, established the space station, and led to the success of the Apollo moon landing program that rendered human activities in space a reality. As humans began to explore outer space, their health in the new space environment became an issue of major concern. During this period, humanity realized that the outer space living environment was quite different from the Earth’s environment. Astronauts living in outer space face many environmental factors, such as partial gravity and outer space radiation, which are not experienced on Earth. With the development of science and technology, humans will be able to travel farther in outer space in the future and will also likely immigrate permanently in partial gravity environments, such as the Moon and Mars. Therefore, during the past 60 years, humans have researched the effects of the outer space environment on human health, including the effects on the human skeletal system.

Different outer space stars have different gravitational pulls. Astronauts aboard the space station, for example, will experience zero gravity, whereas astronauts on the Moon and Mars will experience 16% and 38% of Earth’s gravity, respectively. Despite the fact that humans have successfully conducted animal experiments in space, it has been concluded that it cannot be popularized due to cost and other limitations. To circumvent this constraint, humans have devised and refined a number of gadgets that can simulate gravity. For instance, the tail suspension device can be used to imitate the zero gravity of space (Morey et al., 1979). Similarly, the quadrupedal unloading model can be adjusted to simulate various gravity levels (Mortreux et al., 2018). At the same time, previous research has discovered that altering the tension of the soleus muscle can imitate various amounts of partial gravity levels (Kawano et al., 2004). This renders investigating the effects of gravity on mice and rats on Earth considerably more convenient for researchers.

The human skeletal system is important for human beings, as bones support one’s body weight, provide structural and dynamic stability (gait), and assist in the generation of power by skeletal muscles. It is also a vital system responsible for human hematopoiesis and maintains acid–base balance (Lemann et al., 2003; Taichman, 2005). In view of the importance of bones to human life activities, the effect of the space environment on human bones is worthy of human attention. Many previously published studies have found that the environment of outer space has negative effects on bone health (Stein, 2012; Sibonga, 2013; Smith et al., 2014). Additionally, studies have found that an increase in the level of gravity in space can prevent the decrease in BMD and muscle mass (Shiba et al., 2017).

The skeletal system of the human body is composed of (among others) various bones, each of which has different functions. Some bones mainly protect important organs of the human body such as the skull, whereas some bones are mainly responsible for the body’s weight-bearing and motor functions, such as the lower limb bones. Thus, human bones are classified into non-weight- and weight-bearing bones according to their functions. In weight-bearing bones, different bones and even different parts of the same bone have different weight-bearing conditions.

The association between the site-specificity of BMD alterations and different gravity levels has already been investigated in various previous studies (Maupin et al., 2019; Tominari et al., 2019). Moreover, a previous study summarized the rate of bone loss in various parts of the body of astronauts who have spent a long time in space, emphasizing the site-specificity of BMD reductions, indicating that losses prevail in skeletal regions that are generally weight-bearing on Earth (LeBlanc et al., 2000). If the study’s scope is limited to the lower limb bones from the hip joint to the ankle joint, the response of the lower limb bones in different regions to changes in gravity is also interesting to assess. However, the differences in bone mineral density (BMD) and other bone parameters of the different areas of lower limb attributed to gravity’s effects have been rarely reported in the literature. Likewise, the different effects imposed by the different gravity levels of Mars, Moon, and outer space on the human skeletal system are still unclear.

The knee joint is the largest weight-bearing joint in the human body, and its involvement in the lower limb bones is much more complex and profound than that of the nearby hip and ankle joints. We assume that the partial gravity of outer space has a more profound negative effect on the weight-bearing parts of rat bones. Furthermore, different partial gravity magnitudes have different effects on the same parts of the bones. In this study, we studied the effects of partial gravity on different parts of the lower limb bones of the rat as well as the effects of different partial gravity levels on the limb bone and the tibia’s morphology. This discovery may assist the protection of astronaut bones in future outer space environments.

## Materials and Methods

### Experimental Design and Animal Care

**FIGURE 1:**
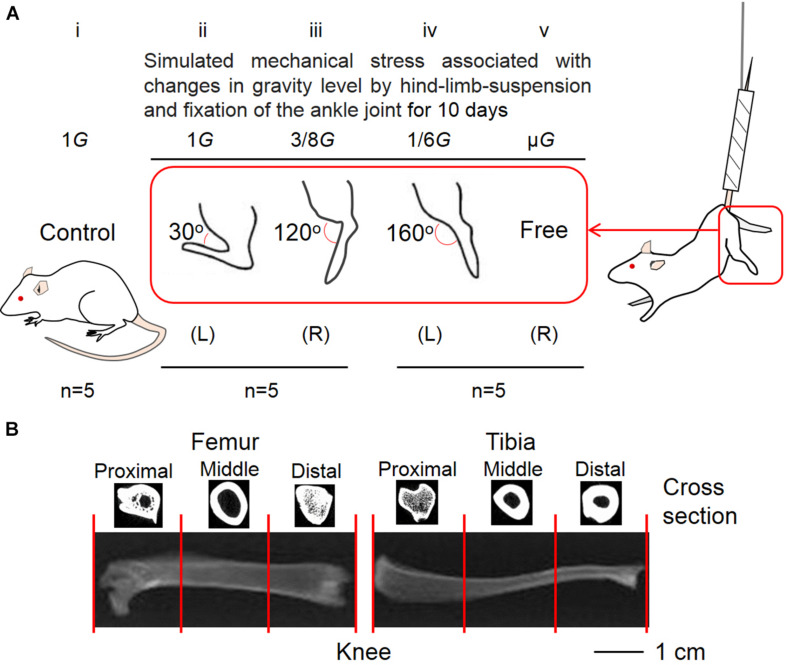
Five experimental groups **(A)** and segmentation of lower limb bones **(B)**. The control **(Ai)** and suspension rat groups had their ankle joints fixed at 30° to simulate the effect of 1*G* experienced on Earth **(Aii)**, 120° to simulate 3/8*G* experienced on Mars **(Aiii)**, and 160° to simulate 1/6*G* of the Moon **(Aiv)**. The ankle joints were not fixed to simulate the μ*G* of the outer space **(Av)**. **(B)** For analysis, the femur and tibia were divided into three equal parts, namely, proximal, middle, and distal positions.

The Animal Care Committee at Doshisha University endorsed the procedure of this experiment (No. A17010). At the beginning of the experiment, 8-week-old male Wistar Hannover rats (Shimizu Laboratory Supplies, Kyoto, Japan) were used. The rats were randomly divided into the control (*n* = 5) (Figure 1Ai) and hindlimb suspension groups (*n* = 10) (Figures 1Aii–v). In five rats that belonged to the hindlimb suspension group, the left ankle joint was fixed at an angle of ∼30° (Figure 1Aii) and the contralateral joint was fixed at ∼120° (Figure 1Aiii) using plaster cast anesthesia with an intraperitoneal injection of sodium pentobarbital (5 mg/100 g body weight). In the remaining five rats, the left ankle joint was fixed at ∼160° (Figure 1Aiv) and the contralateral joints were kept free (Figure 1Av). The ankle joint angles were selected at ∼120° and ∼160° because the soleus stress developments at these angles were approximately equal to 3/8*G* and 1/6*G* (Kawano et al., 2004), which may be equal to those at Mars (3/8*G*) and Moon (1/6*G*) compared with those at ∼30° on the ground in the Earth’s 1*G* setting. To simulate exposure at the μ*G* environment, a suspension with a free ankle joint was conducted.

As mentioned elsewhere, the 10 rats were suspended from the hindlimb for 10 days (Kawano et al., 2004; Ohira et al., 2011). Direct contact between the hindlimbs and the floor and cage wall were prevented. The rats could eat food and drink water freely during this time with their forelimbs. Temperature and humidity with light–dark cycles of 12:12 h in the animal room were maintained during the experimental duration at 23°C and 55%, respectively.

### Bone Sampling and Analyses

The hindlimb bones were separated from the hip joint to the ankle joint. The bones were washed by submerging them in a 5% papain solution (approximately 50°C) under the hood. The femur and tibia were assessed for length and dry weight. The shape of each tibia was also studied, such as the degree of shaft bending and the distal end rotation of the tibia. The area formed by the external curve and the distance indicated by the hatched bars between the proximal and distal edges of the tibia (Figure 4; Ohira et al., 2006) were calculated. Furthermore, by dividing this area by the distance between the proximal and distal sides, the degree of external bending was measured. To approximate the relationship between the middle part of the distal end of the tibia and the tip of the distal end of the fibula, or the anterior tip of the central tibia, two straight lines were drawn (red lines and black spot). To obtain the external rotation of the distal end of the tibia, the angle formed by these lines was determined.

We set the field-of-view of the computer tomography (CT) scan (Latheta LCT-200, Hitachi, Ltd., Tokyo, Japan) to 24 mm and placed the femur in a fixed mold (inner diameter: 12 mm, maximum load: 100 g). Using the program Latheta LCT-200, we picked the scale between the proximal and distal vertices of the femur as the scan measurement range for the entire femur after CT imaging of the femur was shown on the screen. The scan range of the proximal femur was measured by taking the distance from the highest point of the proximal femur to approximately one-third of the length from the highest point of the proximal femur. The middle femur scanning range spanned a distance that began from approximately one-third of its length from the highest point of the proximal femur and ended at approximately two-thirds of the length from the highest point of the proximal femur. Finally, the scanning range of the distal femur spanned a distance that started from approximately two-thirds of the length from the highest point of the proximal femur and ended at the lowest point of the distal femur. The same treatment was performed on the tibia (Figure 1B). Bone parameters (BMD, trabecular BMD, cortical BMD, and cortical thickness) of the whole, proximal, middle, and distal femur as well as tibia were determined with a CT scanner. Meanwhile, the volume of the lower limb bones was also measured by CT.

### Statistical Analysis

Data were analyzed using GraphPad Prism 8 (GraphPad Software Inc., San Diego, CA, United States). All data are presented as mean ± standard deviation. Significant differences among groups (cage control, 30°, 120°, 160°, and free ankle joint) were determined using one-way analysis of variance. Meanwhile the differences between the experiment groups (30°, 120°, 160°, and free ankle joint) and control group as well as the differences among the simulated mechanical stress associated with partial gravity groups (120°, 160°, and free ankle joint) were determined using the Tukey *post hoc* test. Significant differences were determined at the level of *p* < 0.05.

## Results

### Length, Weight, and Volume of Hindlimb Bone

**FIGURE 2:**
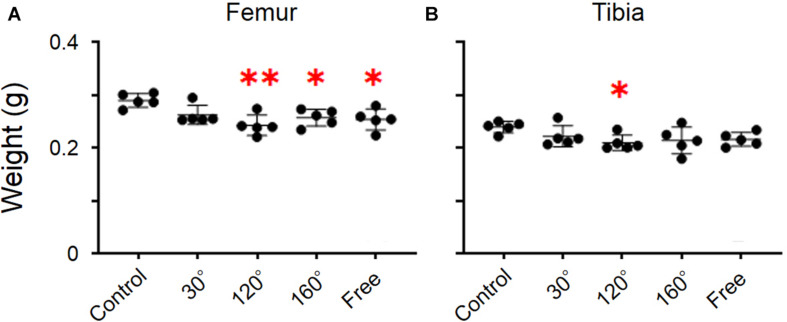
Weights of the femur and tibia. **(A)** Femoral and **(B)** tibial weights [Mean ± standard deviation (SD), ^∗^*p* < 0.05 vs. the control group, ^∗∗^*p* < 0.01 vs. the control group, one-way analysis of variance (ANOVA)].

**FIGURE 3:**
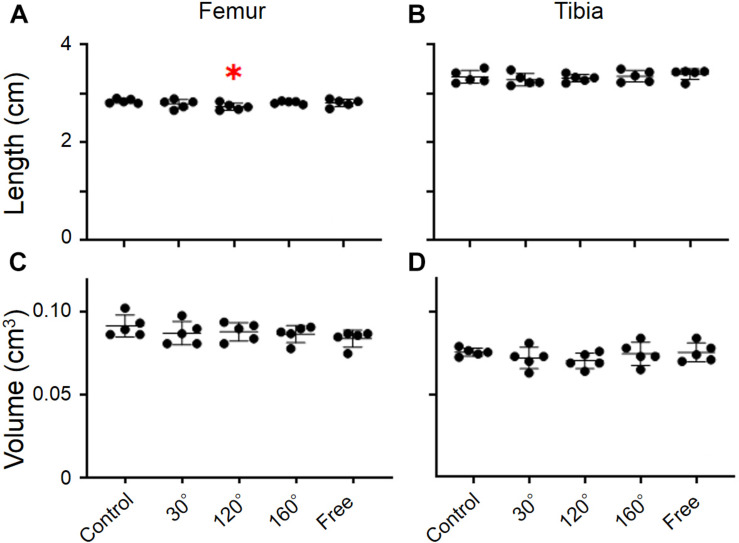
Lengths and volumes of femur and tibia. **(A)** Femoral length, **(B)** tibial length, **(C)** femoral volume, and **(D)** tibial volume [Mean ± standard deviation (SD), ^∗^*p* < 0.05 vs. the control group, one-way analysis of variance (ANOVA)].

The dry weight of the femur was significantly different among the groups (*P* = 0.0055; Figure 2A). The dry weight of 160° and free group was decreased compared with that of the control group (−11.2%, *P* = 0.0258; −12.4%, *P* = 0.0132; Figure 2A). The results revealed that the dry weight of the 120° group in the femur deteriorated more owing to partial gravity effects (−16.2%, *P* = 0.0014; Figure 2A). However, no difference of the dry weight was observed in the 30° group compared with the control group. Regarding the dry weight of the tibia, there were no significant differences among the five groups (Figure 2B). A significant decrease was observed only in the 120° group compared with the control group (−12.4%, *P* = 0.047; Figure 2B). Meanwhile, we did not observe a significant difference in the femoral and tibial weights among the simulated groups of mechanical stress associated with partial gravity. Gravity-associated deterioration in the length and volume of the femur and tibia was not observed, except for that in the femur length of the 120° group compared with that of the control group (Figures 3A–D).

**FIGURE 4:**
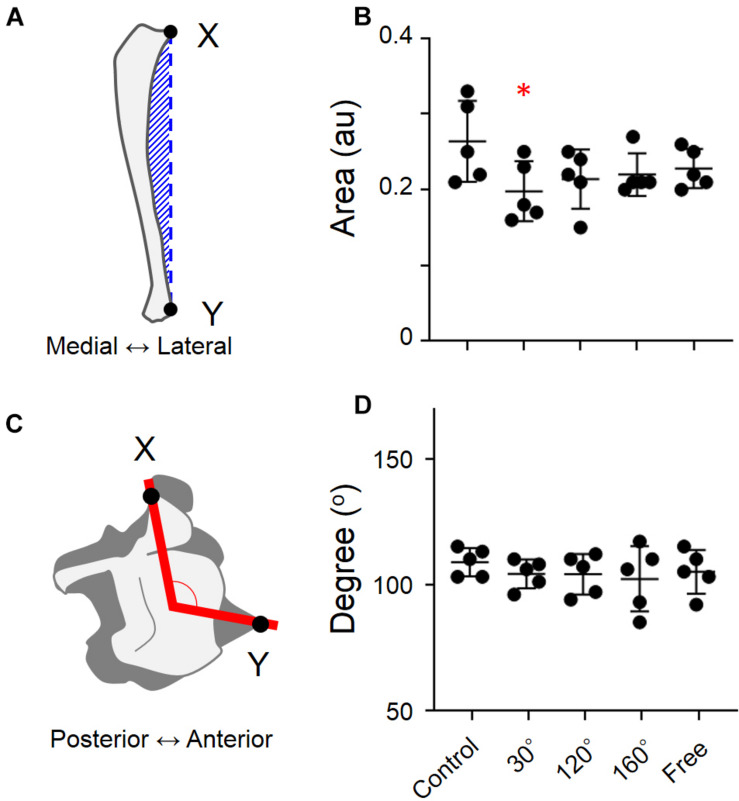
Degree of external curving of tibia and the degree of external rotation of the distal end of tibia in different groups. **(A)** Methods used to estimate the degree of external curving in tibia, **(B)** degree of external curving of tibia, **(C)** degree of external rotation of the distal end of tibia, and **(D)** degree of external rotation of the distal end of tibia [Mean ± standard deviation (SD) ^∗^*p* < 0.05 vs. the control group, one-way analysis of variance (ANOVA)].

### External Bend of the Tibia’s Shaft and Rotation of the Distal End of Tibia

When the simulated groups of mechanical stress associated with partial gravity were compared with the control group, there are no significant differences regarding the degree of external curving of the tibia (Figure 4B). The same result was found in the rotation of the distal end of the tibia (Figure 4D). However, the difference was found when the external curving of tibia of the 30° group was compared with that of the control group (−25.0%, *P* = 0.0449; Figure 4B).

### Bone Parameters of Femur

#### BMD

**FIGURE 5:**
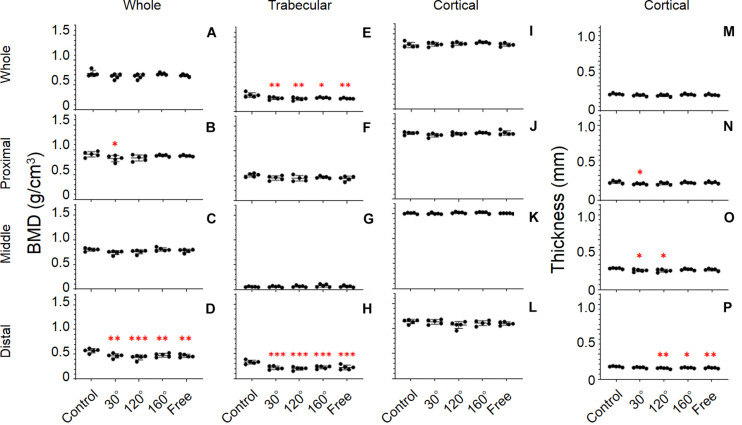
Femoral bone parameters. The bone parameters of different femoral areas of the five tested groups are shown. **(A–D)** BMD, **(E–H)** trabecular BMD, **(I–L)** cortical BMD, and **(M–P)** cortical thickness. **(A,E,I,M)** Whole femur, **(B,F,J,N)** proximal femur, **(C,G,K,O)** middle femur, and **(D,H,L,P)** distal femur [Mean ± standard deviation (SD), *n* = 5. *p < 0.05 vs. the control group, **p < 0.01 vs. the control group, ***p < 0.001 vs. the control group, one-way analysis of variance (ANOVA)].

The response of BMD to the simulated mechanical stress associated with changes in gravity level was evident in the distal femur (*P* = 0.0008; Figure 5D). Compared with the control group, BMD declined in 30°, 120°, 160°, and free group in the distal femur (−17.7%, *P* = 0.0038; −23.8%, *P* = 0.0002; −15.8%, *P* = 0.0097, and −18.0%, *P* = 0.0032, respectively). After comparing the three simulated gravity groups (120°, 160°, and free group) of BMD of the distal area, no significant differences were observed. However, the BMD of the 120° group showed the most profound deterioration in the three simulated gravity groups. Although there are also significant differences in the proximal and middle femur (*P* = 0.0473, and 0.0471), no significant deteriorations were observed in the 120°, 160°, and free groups compared with the control group (Figures 5B,C). No significant differences were observed along the entire femur (Figure 5A).

#### Trabecular BMD

It is worth noting that we observed the most significant deterioration of all the bone parameters in the distal femur (*P* = 0.0001; Figure 5H). The trabecular BMD of 30°, 120°, 160°, and free group all declined significantly compared with that in the control group (−34.6%, *P* = 0.0003; −38.9%, *P* < 0.0001; −31.5%, *P* = 0.0007, and −32.7%, *P* = 0.0005, respectively). The same result appears in the entire femur (*P* = 0.0023; Figure 5E). However, the deterioration of trabecular BMD of the 30°, 120°, and 160°, and free group in the entire femur was less compared with that in the distal femur. Similarly, no significant difference among the three simulated groups of mechanical stress associated with partial gravity was observed in the distal femoral parts and in the entire femur. By contrast, the BMD of the 120° group also yielded the most profound deterioration in the three simulated gravity groups in the distal femoral parts and in the entire femur. Regarding the trabecular BMD of the proximal and middle femur, no significant differences were observed among the five groups (Figures 5F,G).

#### Cortical Thickness

The distal and middle femur yielded significant differences among the five groups (*P* = 0.0043 and *P* = 0.0263; Figures 5P,O). Meanwhile, we also noticed that the difference in the distal femur was much greater than that in the middle femur. In the distal femur, the cortical thickness in the 120°, 160°, and free group cases declined significantly compared with that in the control group (−12.9%, *P* = 0.0012; −8.8%, *P* = 0.0256, and −10.3%, *P* = 0.0085, respectively; Figure 5P). Likewise, the same condition was observed in the middle femur when the 30° and 120° groups were compared with the control group (−9.5%, *P* = 0.0268, and −10.7%, *P* = 0.0113; Figure 5O) as well as in the proximal femur when the 30° group was compared to the control group (−10.1%, *P* = 0.0385; Figure 5N). After the comparison of the three simulated gravity groups in the distal femoral part, no differences were detected. Instead, we obtained the same results as those obtained previously. The 120° group was still the group wherein the most serious deterioration was detected.

#### Cortical BMD

Unlike other bone parameters, the cortical BMD of the whole, proximal, middle, and distal femur was not altered significantly by the simulated mechanical stress associated with changes in gravity level (Figures 5I–L).

### Bone Parameters of the Tibia

#### BMD

**FIGURE 6:**
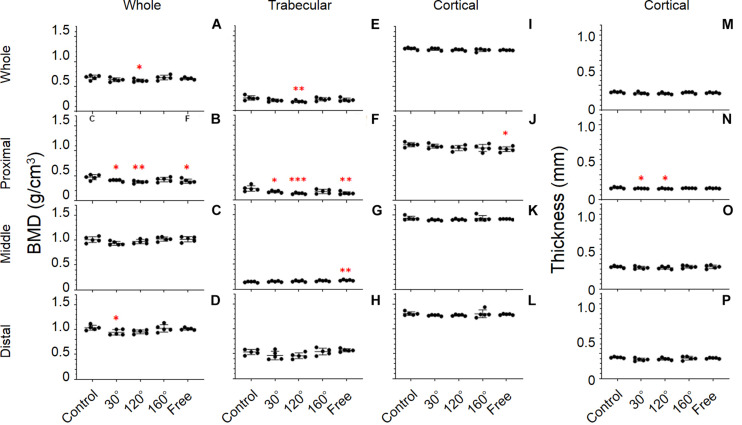
Tibial bone parameters. The bone parameters of different tibial areas of the five tested groups are shown. **(A–D)** BMD, **(E–H)** trabecular BMD, **(I–L)** cortical BMD, and **(M–P)** cortical thickness. **(A,E,I,M)** Whole tibia, **(B,F,J,N)** proximal tibia, **(C,G,K,O)** middle tibia, and **(D,H,L,P)** distal tibia [Mean ± standard deviation (SD), *n* = 5. *p < 0.05 vs. the control group, **p < 0.01 vs. the control group, ***p < 0.001 vs. the control group, one-way analysis of variance (ANOVA)].

The results revealed significant differences in the proximal, middle, and distal tibia (*P* = 0.0071, 0.0386, and 0.0293, respectively; Figures 6B–D). The difference in the proximal tibia was greater than that in the middle and distal tibia. BMD of the 30°, 120°, and free group showed an obvious deterioration in proximal tibia compared with that in the control group (−13.9%, *P* = 0.043; −20.7%, *P* = 0.0022, and −17.1%, *P* = 0.0111, respectively; Figure 6B). The BMD of the 120° group still yielded maximum decreases in the three simulated group of mechanical stress associated with partial gravity. After comparing the three simulated gravity groups (120°, 160°, and free group) of BMD of the proximal area, no significant differences were observed. The BMD of the 120° group also deteriorated in the entire tibia compared with the control group (−9.3%, *P* = 0.0407; Figure 6A). Meanwhile, the BMD of the 30° group in the distal tibia showed significant deterioration (−8.9%, *P* = 0.0343; Figure 6D).

#### Trabecular BMD

A significant difference was observed in the whole, proximal, middle, and distal tibia (*P* = 0.0292, 0.0022, 0.0119, and 0.0314, respectively; Figures 6E–H). Among the whole, proximal, middle, and distal areas, the difference of Trabecular BMD of the proximal tibia is the most conspicuous. In proximal tibia, the 30°, 120°, and free group decreased compared with the control group (−27.3%, *P* = 0.0366; −43%, *P* = 0.001, and −40.2%, *P* = 0.0019, respectively; Figure 6F). Consistent with the previous result, the trabecular BMD of the 120° group in the proximal tibia still declined the most in the three simulated groups of mechanical stress associated with partial gravity. Similarly, no significant difference among the three simulated groups of mechanical stress associated with partial gravity was observed in the proximal tibia. Meanwhile, deteriorations in the trabecular BMD of the 120° group in the entire tibia (−27.1%, *P* = 0.008; Figure 6E) as well as the increase of free group in the middle tibia were observed compared with the control group (+22.7%, *P* = 0.0035; Figure 6G).

#### Cortical Thickness

Results showed significant differences only in the proximal tibia (*P* = 0.0385; Figure 6N). The cortical thickness of the 30°, and 120° group in the proximal tibia decreased, compared with the control group (−6.6%, *P* = 0.044, and −8.0%, *P* = 0.0135, respectively). After the comparison of the three simulated gravity groups in the proximal part, no differences were detected. No significant differences of other areas were observed (Figures 6M,O,P).

#### Cortical BMD

Consistent with the cortical BMD of the femur, there were also no significant differences in the tibia (Figures 6I–L).

## Discussion

In this study, we identified the statistical differences of the lower limb bone parameters in different areas. Furthermore, we noticed that the bone parameters of the distal femur group and proximal tibia, which were close to the knee joints, showed a much more evident deterioration than other areas (Figures 5D,H,P, 6B,F,N). The bones of humans and rats are classified as weight- and non-weight-bearing bones. The article that summarized the previous literature on the BMD of astronauts found that the BMD of non-weight-bearing bones of astronauts in space flights increased or remained unchanged in the skull, sternum, cervical vertebrae, and upper limb bones, whereas the BMDs of the lower limb bones and lumbar vertebrae, which are weight-bearing bones, were progressively reduced (Stavnichuk et al., 2020). These results showed that the effect of the partial gravity on human bones is not uniform, the effect on non-weight-bearing bones is positive or no effect, and the effect on weight-bearing bones is negative. It was also found that BMD was preserved in the upper limbs during space flights but diminished in the lower limbs. Therefore, it is believed that the relationship between BMD changes and position is related to the gravity vector (Oganov et al., 2005). Our data indicated that the BMD, trabecular BMD, and cortical thickness in the cases of the simulated group of mechanical stress associated with partial gravity in these two positions were significantly lower than those in the control group; however, only a few bone parameters in other areas showed significant differences. This discovery shows that even in the lower limb bones, which are weight-bearing bones, partial gravity had a more significant (negative) effect (e.g., knee joint bones). It was reported that 9-week-old C57BL/6 mice were placed in a spacecraft to study gravity’s effect on non-weight- and weight-bearing bones (Maupin et al., 2019). They found that the BMDs of the distal femur and proximal tibia–fibula of the spaceflight group were significantly lower than those of the ground control group. There was no significant difference in the bone parameters in the middle femur and middle tibia–fibula when the spaceflight groups were compared with the ground control group. This research result confirms our conclusion. Our experiment has set up more groups and found that mechanical stress equivalent to partial gravity environment levels can harm different parts of the lower limb bones. More groupings increase the significance of the experimental results.

The human body cells perceive physical forces by attaching receptors to their surfaces and by responding to the applied forces. Bone cells with weight-bearing functions are no exception (DeMali et al., 2014). Bone tissue must be stimulated by mechanical force to balance bone reconstruction and bone resorption, and gravity plays a particularly important role in maintaining balance (Porazinski et al., 2015). To maintain a balanced process, osteoclasts and osteoblasts are the direct participants in bone reconstruction. Subject to the Earth’s gravitational conditions, adult osteoblast and osteoclast activities maintain a balanced state. However, this balance will be disrupted owing to the entirely different outer space environment. Specifically, space activities will cause bone health problems, in which partial gravity plays an essential role (Sides et al., 2005; Grenon et al., 2012; Ma et al., 2019). Some previous studies have found that partial gravity can promote osteoclast production and activity (Vico et al., 1993; Tamma et al., 2009; Blaber et al., 2013; Chatani et al., 2016; Smith, 2020; Stavnichuk et al., 2020). Partial gravity can promote the activation of osteoclast-related signal molecules, thereby leading to increased bone resorption (Yuge et al., 2003; Saxena et al., 2011; Hu et al., 2014).

As the largest weight-bearing joint of the human body, the knee joint has adapted to the Earth’s gravity environment. Once the human body’s and the rat’s gravitational environment changes, the knee joint’s weight-bearing will also change considerably. Therefore, the bone cell response in this part to the force change will also be tremendous. The bone balance maintained by osteoblasts and osteoclasts in this area is also easily affected by partial gravity. This can explain why partial gravity has a more significant influence on these parts’ bone parameters compared with other parts.

Furthermore, our results did not yield any significant differences of cortical BMD (Figures 5I–L, 6I–L), which confirms the results of previous studies that trabecular and cortical bone yielded different responses to partial gravity, and trabecular bone is more sensitive to partial gravity than cortical bone (Krause et al., 2020). Other surveys also found that the cortical outcomes of 2 weeks of hindlimb suspension did not decline compared with that of the ground control group (Krause et al., 2017), but there is an additional cortical bone loss after 3 weeks of hindlimb suspension (Lloyd et al., 2012, 2014). Additional research revealed that trabecular and cortical bone responded differently to partial gravity because trabecular bone has a larger surface area (Jee, 1983), thus indicating that more basic multicellular units can respond to changes in gravity faster and more vigorously (Martin, 2000; Sims and Martin, 2014).

At the same time, after comparing the simulated groups of mechanical stress associated with partial gravity in the distal femur and proximal tibial area, we did not identify significant differences in BMD, trabecular BMD, or cortical thickness among the three groups contrary to our original assumption (Figures 5D,E,H,P, 6B,F,N). The results of our study are similar with the results of the previous study on the effects of different Partial Weight-Bearing (PWB) levels on BMD. Mortreux et al. (2018) randomly divided 38 rats into four groups with different gravity levels in their study: normal loading (PWB100, *n* = 11), 70% of normal loading (PWB70, *n* = 7), 40% of normal loading (PWB40, *n* = 10), and 20% of normal loading (PWB20, *n* = 10). After the use of the new quadrupedal unloading suspension device, rats were suspended to simulate the different partial weight-bearing levels for 14 days, and the trabecular BMDs of the proximal tibia bones were measured with peripheral quantitative CT. This study found that on day 14 of the suspension, there was no statistical difference between the PWB20 and the PWB40 group and between the PWB70 and PWB100 group. The results of this study were similar to ours, and there was no statistical difference in BMD between different simulated gravity levels. Interestingly, we found a consistent tendency among the bone parameters in the distal femur and proximal tibia areas: The 120° group always yielded the most significant difference compared with the control group among the simulated groups of mechanical stress associated partial gravity, whereas the 160° group always yielded the least significant difference among the three simulated partial groups.

Our experimental results showed that the reduction of gravity levels can cause significant differences in femoral weight (Figure 2A). Even still, they had no pronounced effects on the lengths of the femur and tibia (Figures 3A,B). This result is similar to the development of our previous experiment (Ohira et al., 2006) in which there was no significant difference in the length of the tibia owing to the effects of different gravity levels. The tibia’s weight at the 120° level was significantly lesser than that of the control group (1*G*), which is the same as the BMD result of the tibia (Figure 2B). We believe that the bone weight of the femur in the simulated mechanical stress associated with changes in gravity level group and the bone weight of the 120° group in tibia are significantly lower than that of the control group owing to the loss of bone mass in the experiment groups. However, we did not find any statistical difference in the weights among the three experiment groups (120°, 160°, and free group). This shows no differences in the effects of different gravity levels on bone weight.

In contrast to previous research, we found no substantial differences in the degree of external curve of the tibia or the degree of external rotation of the distal end of the tibia among different groups in our study (Figure 4). We believe that there are two possible reasons for this result. The first reason is the rat’s age (in the cases of suspended hindlimb experiments). The rats were suspended from the hindlimb by the tail at postnatal day 4. However, the suspension of the hindlimb began in this experiment when rats were 8 weeks old. The simulated partial gravity’s influence on the morphology of the tibia was more pronounced in the early growth period. The second reason was attributed to the fact that the suspension duration was different. In the previously conducted experiments, the hindlimb suspension time of the rat was approximately 3 months. In this study, the suspension time was only 10 days. Therefore, a suspension time of 10 days may not be enough to cause changes in tibial morphology. Similarly, we believe that these two factors are also responsible for the no statistically significant differences in the bone volumes of the hindlimbs.

Our research is the first to demonstrate that partial gravity does have a stronger effect on rat knee joints than that on other regions of the lower limb bones. This indicates that the influence of partial gravity on bones is related to the weight-bearing capacity of bones. This survey result will provide new ideas and perspectives for subsequent research and can help astronauts to update their protective ideas when performing space missions. However, our study is associated with specific limitations. First, we primarily report the bone parameters and not any micromorphological features on the trabecular and cortical bones when it related to the influence of partial gravity on the different sections of lower limb bones. At the beginning of the establishment of this study, to examine whether the effects of some gravity levels on various parts of the lower limb bones are different, we decided to test the bone parameters first to observe whether the experiment results are consistent with the initial hypothesis. If the experimental results are consistent with the initial hypothesis, we will further study the following objects of different parts of lower limbs: (1) The three-dimensional images (horizontal section, isolated trabecular bone of horizontal section, and vertical section); (2) The static histomorphometry, osteoblast, and osteoclast surface along the trabecular bone; and (3) The mRNA expression of bone formation-related genes and osteoclast genesis-related genes were measured by qPCR. Therefore, further research needs to be based on the present study results of bone parameters. Second, the responses of bones to gravitational unloading are affected by not only the local effects but also the systemic effects. In this study, we could not evaluate these effects separately. However, we consider that the most part of systemic changes in rats, e.g., shifting of body fluid toward head and immobilization stress, in response to hindlimb suspension might have been comparable. In contrast, a part of systemic effects, e.g., alteration of myokines in accordance with atrophy of antigravitational muscles, could have affected contralateral legs, which were adopted as different loading groups. Therefore, we hope that our future research can further explore the changes in micromorphological features, osteoblasts, osteoclasts, and mRNA in the effects of different simulated gravity levels and different parts based on overcoming the current limitations.

## Data Availability Statement

The raw data supporting the conclusions of this article will be made available by the authors, without undue reservation.

## Ethics Statement

The animal study was reviewed and approved by The Animal-Care Committee at the Doshisha University endorsed the procedure of this experiment (no. A17010).

## Author Contributions

YO, TI, and AT participated in the conception and design of the study. DU, HK, and YO performed the hindlimb suspension study. TO performed the cleaning of bones by submerging in a 5% papain solution and analyzed the morphological properties, dry weight, and data analyses. SZ performed CT analysis and analyzed data. SZ, SY, YY, and AT contributed to drafting the article. YO and TI reviewed and edited. All authors contributed to the article and approved the final submitted manuscript.

## Conflict of Interest

The authors declare that the research was conducted in the absence of any commercial or financial relationships that could be construed as a potential conflict of interest.

## Publisher’s Note

All claims expressed in this article are solely those of the authors and do not necessarily represent those of their affiliated organizations, or those of the publisher, the editors and the reviewers. Any product that may be evaluated in this article, or claim that may be made by its manufacturer, is not guaranteed or endorsed by the publisher.
